# Pedagogical knowledge for active-learning instruction in large undergraduate biology courses: a large-scale qualitative investigation of instructor thinking

**DOI:** 10.1186/s40594-018-0112-9

**Published:** 2018-04-12

**Authors:** Anna Jo J. Auerbach, Tessa C. Andrews

**Affiliations:** Department of Genetics, University of Georgia, 120 East Green St., Athens, GA 30602 USA

**Keywords:** Active learning, Cognitive engagement, Teacher knowledge, Pedagogical knowledge, Undergraduate, College instructors, Knowledge for teaching, Teacher noticing

## Abstract

**Background:**

Though active-learning instruction has the potential to positively impact the preparation and diversity of STEM graduates, not all instructors are able to achieve this potential. One important factor is the teacher knowledge that instructors possess, including their pedagogical knowledge. Pedagogical knowledge is the knowledge about teaching and learning that is not topic-specific, such as knowledge of learning theory, classroom management, and student motivation. We investigated the pedagogical knowledge that 77 instructors who report implementing active-learning instruction used as they analyzed video clips of lessons in large active-learning biology courses. We used qualitative content analysis, and drew on cognitive and sociocultural perspectives of learning, to identify and characterize the pedagogical knowledge instructors employed. We used the collective thinking of these instructors to generate a framework of pedagogical knowledge for active-learning instruction in large undergraduate biology courses.

**Results:**

We identified seven distinct components of pedagogical knowledge, as well as connections among these components. At the core of their thinking, participants evaluated whether instruction provided opportunities for students to generate ideas beyond what was presented to them and to engage in scientific practices. They also commonly considered student motivation to engage in this work and how instruction maximized equity among students. Participants noticed whether instructors monitored and responded to student thinking in real-time, how instruction prompted metacognition, and how links were built between learning tasks. Participants also thought carefully about managing the logistics of active-learning lessons.

**Conclusions:**

Instructors who report using active-learning instruction displayed knowledge of principles of how people learn, practical knowledge of teaching strategies and behaviors, and knowledge related to classroom management. Their deep knowledge of pedagogy suggests that active-learning instruction requires much more than content knowledge built through training in the discipline, yet many college STEM instructors have little or no training in teaching. Further research should test this framework of pedagogical knowledge in different instruction contexts, including different STEM disciplines. Additional research is needed to understand what teacher knowledge is critical to effective active-learning instruction and how the development of this knowledge is best facilitated. Achieving widespread improvement in undergraduate STEM education will likely require transforming our approach to preparing and supporting undergraduate instructors.

## Background

Active-learning instruction in undergraduate STEM courses can be highly effective in facilitating the development of conceptual understanding and scientific thinking skills (e.g., Crouch and Mazur 2001; Deslauriers et al. 2011; Freeman et al. 2014). Importantly, active learning may also improve the diversity of STEM graduates because it can disproportionately benefit students belonging to underrepresented groups (e.g., Springer et al. 1999; Haak et al. 2011; Eddy and Hogan 2014). However, the student outcomes that instructors achieve using active learning vary significantly (e.g., Pollock and Finkelstein 2008; Andrews et al. 2011). Instructors often use active-learning strategies differently than intended by developers (e.g., Turpen and Finkelstein 2009; Dancy et al. 2016), and relatively small decisions about how to implement a teaching strategy can have substantial impacts on student learning (e.g., Smith et al. 2009; Knight et al. 2013). An instructor’s knowledge influences how he or she plans and implements active-learning instruction, ultimately affecting student learning (e.g., Hill et al. 2005; Park et al. 2011; Sadler et al. 2013; Blömeke et al. 2015; Stains and Vickrey 2017). Therefore, there is a critical need to better understand the knowledge that is important to effective active-learning instruction. We will be better equipped to design evidence-based support for college STEM instructors to achieve the benefits of active learning for their students once we are armed with this understanding.

### Guiding theoretical frameworks and prior research

**Fig. 1:**
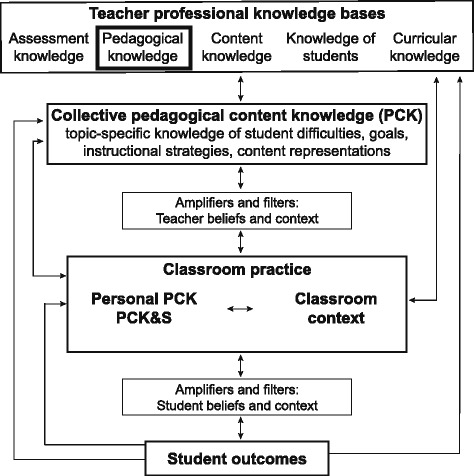
Model of teacher professional knowledge and skill adapted from Gess-Newsome (2015). This model, which emerged from a meeting of researchers studying pedagogical content knowledge, guided our work. The box around pedagogical knowledge highlights the theoretical contribution this work aims to make—to elaborate on our understanding of this knowledge base for active-learning instruction in large undergraduate biology courses

We review the theories and empirical work that guided our research aims and approach and highlight the novel contribution made by this study. There is a rich history of investigating teacher knowledge among K12 instructors. Most teacher knowledge research since the late 1980s has followed from Shulman’s delineation of domains of teacher knowledge and his emphasis on pedagogical content knowledge (Shulman 1987). Pedagogical content knowledge (PCK) is the knowledge of teaching and learning that is specific to a topic (e.g., natural selection, forces and motion, averages) and grade-level (Gess-Newsome 2015). How PCK is conceptualized and studied has been the focus of ongoing scholarly debate. In 2012, PCK researchers gathered for a working summit with the goal of resolving persistent divergences in the field (Carlson et al. 2015). One outcome of this meeting was a consensus model of teacher’s professional knowledge and skill, including PCK (Fig. 1). This model guided our research.

This model of teacher’s professional knowledge and skill unpacks the relationship between teacher knowledge and student learning by recognizing many components of teacher knowledge and multiple factors that influence teaching and learning (Fig. 1, Gess-Newsome 2015). The top of the model depicts generalizable knowledge bases for teaching. Those shown in Fig. 1 were included in the first publication of this model but were not presented as exhaustive (Gess-Newsome 2015). One example of other potential knowledge bases is general and topic-specific technological knowledge, which could support technology integration in teaching (Mishra and Koehler 2006). This paper focuses on pedagogical knowledge, so we have highlighted this with a dark outline (Fig. 1). Pedagogical knowledge (PK) is the knowledge of pedagogy that is potentially generalizable across topic and even discipline. Much less research has examined PK compared to PCK, so it is not well-defined. It may include knowledge of theories of learning, general principles and approaches to instruction and assessment, lesson structure, classroom organization and management, student motivation, and other knowledge of learners (e.g., Shulman 1987; Grossman and Richert 1988; Morine-Dershimer and Kent 1999; König et al. 2014).

The next parts of the model of teacher professional knowledge and skill represent theoretical contributions of the consensus model. An ongoing area of debate is whether PCK should be considered static knowledge possessed by teachers independent of context or whether it is context-dependent and embedded in the actions of teaching (e.g., Depaepe et al. 2013). The consensus model includes both but clearly distinguishes between them. Collective PCK is a public understanding generated by research and best practice and is a canonical and static body of knowledge available for study by teachers (Gess-Newsome 2015). The consensus model calls this “topic-specific professional knowledge,” but we use “collective PCK” like Smith et al. (2016) because it is easier to understand in the context of prior PCK research. Personal PCK and personal PCK&S (pedagogical content knowledge and skill) are privately held rather than public and are contextualized within a particular classroom context at a particular time (Gess-Newsome 2015). Distinguishing personal from collective knowledge recognizes that real contexts include uncertainty, complexity, and uniqueness and thus cannot be addressed purely by applying static knowledge (Schön 2001). Personal PCK is applied in the planning of and reflection on instruction and includes reasons behind instructional plans. It is very similar to what Schön (1983) called reflection-*on*-action and what Alonzo and Kim (2016) called declarative PCK. Personal PCK&S occurs in the act of teaching as instructors monitor what is occurring and make decisions about how to adjust their instruction. Alonzo and Kim (2016) refer to this as dynamic PCK and Schön (1983) refers to it as reflection-*in*-action. Another major theoretical contribution of the consensus model is moving teacher beliefs outside the conceptualization of teacher knowledge. The model views teacher’s beliefs about teaching, learning, and students as a filter or amplifier between knowledge bases and instructional practice (Gess-Newsome 2015).

A theoretical perspective called teacher noticing also informed our research. Teacher noticing is considered a component of teacher expertise (Sherin et al. 2011). Teacher noticing involves an ability to pay attention to, reason about, and respond to important events in real time while teaching (e.g., van Es and Sherin 2008; Sherin et al. 2011). This way of thinking about teacher knowledge recognizes that classrooms are multidimensional and unpredictable and that many things occur simultaneously (Sherin et al. 2011). The model of teacher professional knowledge and skill is not described in relation to teacher noticing (Gess-Newsome 2015), but we see an overlap between PCK&S and teacher noticing. Both focus on thinking and decision-making that occur in real time while teaching and in response to careful observation of student thinking (van Es 2011; Gess-Newsome 2015).

Both the consensus model of teacher knowledge and skills and the construct of teacher noticing provide a strong grounding for our work but also fall short in important ways. Neither focus on pedagogical knowledge nor the role of this knowledge in instructional practices and student outcomes. Additionally, pedagogical knowledge is defined only broadly (if at all), and the nature of this knowledge remains unexplored. PCK is defined as being both collective and personal, but prior work is silent on the nature of pedagogical knowledge. Yet a major difference between a traditional lecture and active-learning instruction is the pedagogy, so this may be an important knowledge base for instructors using active learning. Our work takes a first step in filling these gaps. We aim to characterize pedagogical knowledge for active-learning instruction in large undergraduate biology courses.

Drawing on prior teacher noticing research, we used a lesson analysis approach to elicit teacher knowledge in this study. Lesson analysis involves showing an instructor a short video clip of a classroom and asking them to analyze what is occurring. This approach has been used repeatedly to study what teachers notice and to assess teacher knowledge (e.g., Kersting 2008; Kersting et al. 2012; Santagata and Yeh 2013; Kaiser et al. 2015). Importantly, the ability of the teachers to analyze video clips of lessons predicts teaching quality and student learning (van Es and Sherin 2008; Kersting et al. 2012; Santagata and Yeh 2013). We used prior work as a guide in designing a lesson-analysis survey. We used 3- to 5-min videos, like Kaiser et al. (2015), so that instructors saw the arc of an entire activity. We used general prompts for each video, like Kersting et al. (2012), rather than more specific prompts to avoid cueing instructors to aspects of instruction we deemed important. It was important to avoid influencing what participants noticed as they analyzed lessons because we aimed to identify the pedagogical knowledge they naturally drew on to critique a lesson.

### Prior research on teacher knowledge among undergraduate STEM instructors

Investigations of teacher knowledge among college STEM instructors are sparse, but the few existing studies point to an important role for PK and PCK in evidence-based teaching. Semester-long studies of college mathematics instructors adopting an inquiry-based curriculum for the first time revealed that they lacked awareness of likely student difficulties with specific topics (i.e., PCK). This left them floundering to make sense of students’ ill-formed reasoning in-the-moment while facilitating discussions and struggling to recognize how the ideas that students contributed were relevant to lesson goals (Wagner et al. 2007; Speer and Wagner 2009; Johnson and Larsen 2012). We previously studied the knowledge used by 14 expert and 29 novice active-learning instructors as they completed the same lesson-analysis survey used in this study (Auerbach et al. 2018). Experts were better able to support their lesson analyses with reasoning. They also more commonly considered how instructors hold students accountable, topic-specific student difficulties, whether the instructor elicited and responded to student thinking, and opportunities students had to generate their own ideas (Auerbach et al. 2018). This work primarily made quantitative comparisons among experts and novices and fell short of richly characterizing the knowledge used by active-learning instructors.

In this study, we investigated the thinking of a sample of 77 instructors to address this research question: What *pedagogical knowledge* do active-learning instructors who teach large college biology courses use to critically analyze active-learning lessons? Due to the dearth of prior research on pedagogical knowledge used by college STEM instructors, we aimed to thoroughly characterize the knowledge that participants used and to develop a framework for organizing this knowledge. We are beginning to unpack the pedagogical knowledge component in the consensus model of teacher’s professional knowledge and skill (Fig. 1), and to do so within our instructional context of interest. In addition, we see value in the framework produced by this work for individuals who design and implement teaching professional development for college biology instructors because it suggests potential learning objectives for instructors who want to improve their use of active learning in large classes. This is but a first step in understanding what knowledge is necessary for effective active-learning instruction. Future research will need to directly examine the pedagogical knowledge that instructors rely on in their own teaching and how this knowledge is related to instructional practice and student outcomes.

The framework emerging from this study represents the collective knowledge of 77 college biology instructors who use active-learning instruction in large courses. Large courses are ubiquitous in undergraduate STEM education and may require specialized pedagogical knowledge because they pose unique instructional challenges. We present the participants’ collective expertise, as well as variation across individuals.

### Defining active-learning instruction

Studying pedagogical knowledge for active-learning instruction requires clearly defining what we mean by active-learning instruction. Previously, active learning has been so poorly defined that some have proposed we do away with the term entirely (Cooper 2016). However, we contend that active learning continues to be a useful construct. Prominent studies have greatly increased awareness of the term “active learning” among college STEM faculty (e.g., Freeman et al. 2014), creating an opening for serious discussions of teaching and learning. Existing empirical and theoretical work provides a strong foundation for defining active-learning instruction, including work grounded in cognitive and sociocultural^1^ perspectives of learning.

The ICAP framework, which has its roots in a cognitive perspective of learning, reframes active learning in terms of the level of cognitive engagement asked of students in learning tasks. ICAP refers to Interactive, Constructive, Active, and Passive levels of cognitive engagement (Chi and Wylie 2014). The ICAP framework articulates the overt behaviors of students that we will observe with different levels of cognitive engagement and the learning and cognitive outcomes we can anticipate from these levels of engagement (Table 1). The ICAP framework is firmly grounded in constructivism, which contends that knowledge results from intentional, active, and ongoing construction on the part of the individual (Piaget 1985).

**Table 1:** Four levels of cognitive engagement in ICAP framework, described by observable student behavior and expected learning outcomes (adapted from Chi and Wylie 2014)

Mode	Interactive	Constructive	Active	Passive
Student behavior	Two or more learners discuss, with each taking turns and generating outputs that go beyond the information that has been presented in instructional materials (e.g., defending and arguing a position)	Learners generate outputs that go beyond the information that has been presented in instructional materials (e.g., drawing a concept map, solving a new problem)	Learners make physical manipulations without adding new knowledge (e.g., taking verbatim notes)	Learners receive information (e.g., listening)
Learning outcomes	Deepest understanding, potential to innovate new ideas, interpretations, products.	Deep understanding, potential for transfer to new contexts	Shallow understanding, potential for transfer to very similar contexts	Minimal understanding, potential for knowledge recalled verbatim and in identical context

Empirical studies suggest that engaging in interactive tasks promotes more knowledge acquisition than engaging in constructive tasks, which promote more knowledge acquisition than engaging in active tasks, which promote more knowledge acquisition than engaging in passive learning tasks: I > C > A > P (Chi 2009; Menekse et al. 2013; Chi and Wylie 2014; Chi et al. 2016). For example, if we consider the use of concept maps, generating or correcting concept maps led to greater knowledge acquisition than copying a concept map, reading a concept map, or constructing a map by selecting the elements from an instructor-generated list (e.g., Chang et al. 2002; Schmid and Telaro 1990; Yin et al. 2005). Additionally, collaboratively building a concept map promoted greater conceptual knowledge acquisition than building one alone (Czerniak and Haney 1998). If an aim of active-learning instruction is deep conceptual understanding, then we should limit it to the interactive and constructive modes of cognitive engagement. Collectively, we can refer to these as “generative” cognitive work because both involve learners generating outputs beyond what has been presented to them (Table 1). We began our study defining active learning solely using this definition. However, initial data analysis suggested that this definition did not fully capture how our participants defined active-learning instruction.

One shortcoming of the ICAP framework is that it draws solely on a cognitive perspective of learning. Learning scientists propose that a sociocultural perspective of learning is also necessary to explain empirical data about how people learn (e.g., Sfard 1998; Vosniadou 2007). In a cognitive perspective of learning, knowledge is seen as an entity that a person can possess and the goal of learning is knowledge acquisition (Sfard 1998). In contrast, a sociocultural perspective of learning sees learning as legitimate participation in a community of practice (Lave and Wenger 1991). In STEM, the community of practice may be the discipline (e.g., physics, mathematics) or something broader (e.g., science) or more narrow (e.g., optics). From a sociocultural perspective, the goal of learning is the adoption of the practices, norms, and discourse of the community in order to fully participate in it (Sfard 1998). Learning is inextricably linked to the context and culture in which it takes place (i.e., contextualized and culturally embedded) and occurs among individuals in the community of practice (Sfard 1998; Vosniadou 2007).^2^

Instruction grounded in a sociocultural perspective engages students in tasks that replicate the discourse and practices of the community and creates opportunities for learners to contribute to the community (Wegner and Nückles 2015). Recent calls for reform in undergraduate STEM education have stressed the need for greater incorporation of opportunities to engage in the practices of sciences (e.g., American Association for the Advancement of Science 2011; Cooper et al. 2015), and this is also aligned with calls for change in K12 STEM education (National Research Council 2012). Being able to think like a scientist and to engage in the practices of science may help students see themselves as scientists and be recognized by others as scientists (i.e., identify as scientists), ultimately contributing to their motivation and persistence in STEM (Seymour and Hewitt 1997; Carlone and Johnson 2007; Graham et al. 2013). Thus, a sociocultural perspective of learning encompasses both cognitive and affective components of learning in STEM.

Ultimately, we drew on both the ICAP framework and a sociocultural perspective of learning to define active-learning instruction within the emergent framework of pedagogical knowledge as legitimate generative cognitive work, in which learners (a) generate outputs that go beyond what has been explicitly presented in instructional materials (i.e., generative work) and (b) belong to a community engaged in the practices and discourse of the discipline (i.e., legitimate work). This definition of active-learning instruction both emerged from and guided our iterative qualitative analysis of participants’ pedagogical knowledge.

## Methods

### Participant identification and recruitment

We studied undergraduate biology instructors who taught large (50+ students) biology courses and who described using active-learning instruction. We aimed to capture individual variation across a range of instructors who consider themselves to be active-learning instructors, so we included instructors who described using strategies in which the instructor stopped lecturing and students worked during class. Capturing this variation is important to determine if the components of pedagogical knowledge are common across college biology instructors, or whether there is variation in pedagogical knowledge.

We used three approaches to identify potential participants. We sent a query to the listserv of the Society for the Advancement of Biology Education Research (SABER) asking for help identifying active-learning instructors. We contacted SABER members because this group includes individuals who have led teaching professional development and individuals who conduct education research in their own and others’ classrooms. Therefore, they have numerous opportunities to meet active-learning instructors. Additionally, most members of SABER have positions in life science departments, providing ample chances to interact with colleagues about teaching and become aware of who is using active learning (e.g., Andrews et al. 2016). Our post to the listserv included a link to an online survey to easily share names of active-learning instructors. We identified and contacted 141 potential participants using this approach.

Next, we contacted individuals involved with initiatives aiming to improve undergraduate biology education. The National Science Foundation (NSF) has funded such projects through current and former programs. Descriptions of all funded projects are publically available in a searchable list on the NSF website. We identified principal investigators (PIs) for projects funded through Improving Undergraduate STEM Education (IUSE) and Widening Implementation and Demonstration of Evidence-Based Reforms (WIDER) programs. We also identified PIs who may still have active projects as evidenced by their attendance at the 2016 Envisioning the Future of STEM Education (EnFUSE) conference hosted by the American Association for the Advancement of Science and the NSF. These PIs could have been funded through Course, Curriculum, and Laboratory Improvement (CCLI) and Transforming Undergraduate Education in STEM (TUES) programs. We used publically available project descriptions to identify projects related to undergraduate biology and active learning. We asked each PI about their own experience using active-learning instruction as well as instructors they had worked with as part of their project. We identified and contacted 145 potential participants using this approach.

Our last approach to identify potential participants involved contacting organizations that offer professional development training to college biology instructors who use or are interested in using active-learning instruction. We used publicly available lists of participants to collect contact information. In other cases, organizers provided contact information for former participants of teaching professional development. We identified and contacted 55 potential participants using this approach.

We contacted each potential participant by email, briefly explained the purpose of our study, and asked if they were available for a short (< 10 min) meeting to conduct a screening interview. We sent a maximum of four follow-up emails to schedule this interview. We scheduled phone calls or virtual meetings to conduct the screening interviews with all potential participants who responded to our emails. The full screening interview protocol is in the Supplemental materials of Auerbach et al. (2018). We used screening interviews to ask instructors how long they had been using active-learning strategies and what active learning looked like in their classroom. We did not define active learning in these interviews and aimed to recruit instructors who described some instruction in which they stopped lecturing and students worked. Hereafter, we refer to these participants as “active-learning instructors.” We also confirmed that potential participants currently taught biology courses with 50 or more students. Out of 341 potential participants emailed, 141 responded and participated in a screening interview, and we invited 109 to complete the survey. Eighty-one faculty completed the survey, representing 74% of those invited following a screening interview. We later omitted four due to inconsistencies between survey and interview responses or incomplete survey responses.

We targeted two particular groups in our data collection because we also used the data for a companion study that made quantitative comparisons (Auerbach et al. 2018). We preferentially sampled novices, who reported using instruction in which they stopped lecturing and students worked for fewer than 4 years and had no evidence of their own effectiveness or a reflective teaching practice. We also aimed to recruit experts, who reported engaging students in generative cognitive engagement (Table 1, Chi and Wylie 2014) for four or more years and had evidence of robust student learning gains and/or a systematic reflection practice. Screening interviews and other data collection allowed us to make these designations. More details about the criteria for experts and novices can be found in Auerbach et al. (2018). In brief, experts met one or both of the following criteria: (A) had used a pre- and post-test design with a research-based instrument to collect data on student learning and achieved an effect size of 0.8 or higher and/or (B) described a highly reflective and systematic approach they used to monitor student thinking or student learning that involved collecting data, comparing data to learning objectives, and changing instructional practices iteratively over many semesters. Both criteria were necessary because not all learning objectives are easily assessed with existing research-based instruments, and pre- and post-testing is not a standard practice in undergraduate biology education. We determined whether criterion A was met by asking participants to share de-identified pre- and post-test data they had collected in a large active-learning class. We calculated the effect size using these data. We determined whether criterion B was met in the screening interview by asking questions about they gathered data about what was and was not working in their classroom.

### Data collection and survey

We developed and iteratively refined a lesson-analysis survey to elicit teacher knowledge. Videos of authentic active-learning lessons in large (50+ students) undergraduate biology courses served as stimuli, followed by writing prompts that asked participants to evaluate the lesson and make suggestions for improvement. We refined initial versions of the lesson-analysis survey by collecting and analyzing responses from instructors with varying levels of active-learning expertise and gathering expert feedback. Step-by-step details of this development process are in Auerbach et al. (2018). The final version of the survey, which was administered online, included three video clips that were 3 to 5 min long. Each included footage of the instructor and the students working. Participants watched a video clip and then answered questions about that video before moving to the next video clip. After each of the first two videos, instructors responded to two written prompts:

1. What was effective and why did you think it was effective? Please use complete sentences.
2. What needs to be improved and why? How would you do it differently? Please use complete sentences.

After the third video, we asked the instructors to respond to question one because question two did not uncover additional thinking from participants in data collected in the development and refinement of videos and prompts within the lesson-analysis survey.

We filmed full class periods to create footage for the video clips and selected segments of the class to use as stimuli. We selected segments of instruction that showed more than one type of instructional activity (e.g., lecturing, individual student work, and small-group work), for which we had high-quality video and audio of both students and the instructor, and that could be turned into short, self-contained clips with limited editing. Editing introduces the perspective of the editor, and we aimed to create clips that were as authentic as possible. We selected clips to show a range of levels of cognitive engagement and types of student work. We also considered gender diversity across instructors. We briefly describe the instructional approaches in each video clip to provide context for the results.

In the first video clip, the instructor described traits that humans and other great apes share and traits that distinguish humans. She used a common student question to frame an activity: Did humans evolve from chimpanzees? She told students that humans did not evolve from chimpanzees and asked them to work in groups and use a projected primate phylogeny to determine why the answer to the question was no. The instructor then asked if anyone could provide the reasoning for why the answer to the question was no. A student volunteered an answer and instructor followed up with a detailed explanation of the reasoning.

In the second video clip, the instructor began by briefly defining genetic drift. He introduced a worksheet activity that required students to roll a die and graph allele frequency changes based on the number on their die to mimic random changes in allele frequency. Students worked on the activity, and the instructor circulated answering multiple questions about the logistics of activity. After some student work time, the instructor demonstrated to the students what they should be doing using a document camera. He then circulated the classroom again, continuing to answer questions about the logistics of the activity. The instructor asked the students if they needed more time, learned that they did, and then moved on to immediately summarize the activity rather than allowing more time for students to work.

In the third video, the instructor showed the students data they had previously examined in a breakout session. She asked the students to explain the data they had seen before and provided guidance about how to monitor their own thinking as they answered this question. She also asked the students to examine the data they had not previously considered and to explain why the data was important to the study. She asked the students to write alone and told them they would talk in groups and as a whole group later. After quietly writing down their thinking, the students discussed their answers in small groups. The instructor circulated the classroom and responded one-on-one to a student’s question about the content, asked a follow-up question, and prompted the student to check in with a neighboring student about their thinking. The instructor asked a few students if they needed more time and determined they had had enough time. She then started a whole-class discussion by asking for a volunteer who had yet had a chance to share their thinking that day.

In addition to collecting data using the lesson-analysis survey, we collected data about relevant professional experiences in the online survey. We also asked the participants about their teaching experience, experience with teaching professional development, and experience with education research. We collected CVs to confirm reports about education research. We offered all participants a $25 gift card as an incentive for survey completion. This study received Institutional Review Board approval prior to data collection, under protocol #00002116.

### Participant demographics

Our sample consisted of 77 college biology instructors with a range of experience and expertise with active-learning instruction, including 14 who we considered experts, 29 novices, and 34 who did not meet novice or expert criteria. Seventy-five percent of these instructors identified as female. Racial and ethnic diversity was limited; 1% of participants identified as Hispanic and 10% identified with any race besides white, including participants who identified as African American/Black, Asian, and American Indian or Alaskan Native. The mean number of students per section in the largest undergraduate course taught by each participant was 233 (SD = 145) students. Participants had taught college biology courses for a median of 16.5 (SD = 14.3) terms (i.e., semesters or quarters), including 32 instructors who had taught for more than 20 terms. Participants had used active learning in large courses for a median of four (SD = 3.4) years, and 27 instructors reported five or more years of experience. More than half of participants (*n* = 50, 65%) had published discipline-based education research. Lastly, 85% of participants (*n* = 65) reported participating in 40 or more hours of teaching professional development.

### Qualitative data analysis

Our data analysis aimed to fully characterize the thinking of our participants, to organize their ideas and reasoning into a framework of pedagogical knowledge for active-learning instruction in large courses, and to describe the variation across participants. We used the participant responses to the lesson-analysis survey as data for this study. We imported all responses into Atlas.ti to organize and facilitate qualitative content analysis. Our qualitative analyses were collaborative and highly iterative. We coded all data in teams of at least two researchers and discussed coding decisions until we reached consensus. We completed all coding blind to the identity of the participant.

We aimed to discover a framework of pedagogical knowledge for active-learning instruction within our participants’ thinking, while also grounding our analysis in relevant prior work. Our analysis drew on essentials of grounded theory, including remaining open to ideas emerging from the data, writing detailed analytic memos throughout analysis, using constant comparative analysis, progressing from initial codes to conceptual categories to diagramming the relationships among categories, and finally generating a comprehensive theory (e.g., Charmaz 2006; Birks and Mills 2011). Our approach differed from a grounded theory approach in two important ways. First, we did not use theoretical sampling to seek specific new data (Charmaz 2006). Second, the ICAP framework and the sociocultural perspective of learning provided guiding lenses for analyzing our data. Therefore, our final framework uses our data to build on existing theory, rather than generating entirely new theory (Charmaz 2006).

We began our analysis with initial coding that aimed to catalog every idea expressed by our participants (Charmaz 2006). We read the participants’ responses, identified each section of text that communicated a distinct idea or ideas, and assigned code(s) that captured these ideas (Birks and Mills 2011). We used some codes that were generated during the development and refinement of the videos and prompts of the lesson-analysis survey and some from prior theory. For example, we began with codes for “active” and “generative” cognitive engagement, which follow from the ICAP framework, and codes for scientific practices, which follow from a sociocultural perspective. Most codes emerged from our data (Charmaz 2006). We began to group the codes into tentative groups to represent broader conceptual categories during initial coding.

We engaged in constant comparative approaches throughout our data analysis, resulting in many iterations of defining and revising codes, conceptual categories of codes, and the framework (Birks and Mills 2011). We compared quotes within and between codes to refine the boundaries and the interrelatedness of the codes. On multiple occasions, both researchers independently grouped codes into categories, presented the categories to each other in concept maps, and discussed our thinking. We drew on participants’ rationales to map relationships among codes. We also examined which codes co-occurred in the same segments of text to provide additional information about how codes related to each other. Discussions about how to group codes into categories occurred throughout the analysis process. Each time we revised the grouping of codes, we re-read every quote within each code under consideration. We also wrote analytic memos to capture our thinking and how it progressed throughout this process. Gradually, our analysis moved toward higher levels of abstraction that ultimately allowed us to generate a framework.

Generating and refining a framework of pedagogical knowledge for active-learning instruction involved determining which codes were relevant to our goals and which were irrelevant. We omitted three types of codes from the final framework. First, we excluded codes that were not about generative cognitive work, including a code about lecturing, two codes about “active” cognitive engagement, and a code about superficial aspects of instructional materials and instructor delivery. For example, one quote that we coded as being about active cognitive engagement was, “Students engaged in activity, not just listening to a lecture on allele frequencies.” There is not enough information in this quote to conclude that the participant was considering the cognitive engagement of students. Second, we omitted codes for quotes that were too vague for us to make a confident judgment about their content. Most commonly, these quotes described an observation without any indication of why the participant was paying attention to what they had noticed. For example, one participant wrote “noted African American students sitting together.” This participant might have been thinking about equity in the classroom or course climate or something else. Their words do not provide enough information for us to make this determination. Third, we omitted two codes that accounted for quotes revealing topic-specific knowledge of teaching and learning (i.e., PCK), because the focus of this work is on knowledge of teaching and learning that is generalizable beyond topic.

Although we began grouping codes into conceptual categories during our initial coding, this became our primary focus as analysis progressed. We aimed to identify distinct pedagogical components about which active-learning instructors think. As part of this process, we expanded our literature review and revisited research to better understand existing theory and research related to categories emerging from our analysis. Some components are unitary, and others have subcomponents because participants reasoned about different aspects of the same overarching pedagogical component. The reasoning participants provided for what they attended in lessons was especially important for grouping codes into components and essential for identifying the connections among components.

Elucidating the connections among pedagogical components began early in the analysis, continued throughout, and ended our framework refinement process. The first framework we generated included our impressions of the connections among components, which were formed based on months of being immersed in the data. We represented these connections as arrows between components showing direction and labeled to indicate the nature of the relationship. Next, we treated each arrow as a hypothesis and systematically investigated our data to determine if they supported each relationship. We read every quote within each component again, paying special attention to relationships among components. This process allowed us to determine which arrows were supported by our data and to generate a summary statement of the relationship indicated by arrow labels.

We aimed to reduce the influence of any one video on our final organizing framework of pedagogical knowledge. The videos that we selected as stimuli influenced the pedagogical components participants had the opportunity to notice and analyze, which could influence the final framework generated by our data. We investigated whether any components of the final framework were closely related to a single video in the lesson-analysis survey. For two components and one subcomponent, ~ 86% of quotes were about a single video. We considered these parts of the framework to be video-specific and have indicated this with dashed boxes in the framework. No more than 60% of quotes focused on a single video for any other component or subcomponent in the framework.

## Results

We present a framework of college instructors’ pedagogical knowledge for active-learning instruction in large courses. This framework consists of seven pedagogical components that emerged from our analysis of what participants noticed and how they reasoned about what they noticed as they analyzed of active-learning lessons. We present an overview of the framework with particular focus on the connections among components, rich qualitative descriptions of each component, and a summary of the variation in knowledge across participants.

### Overview of the framework of pedagogical knowledge for active-learning instruction

**Fig. 2:**
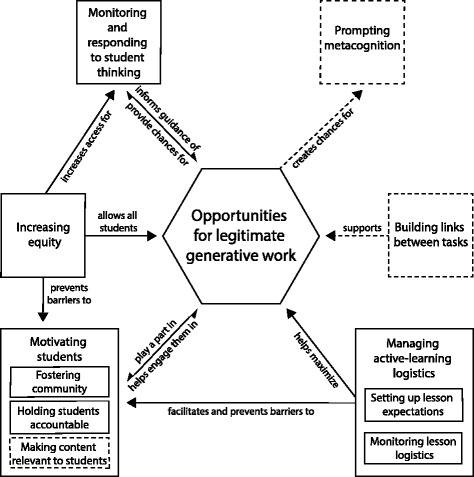
Framework of pedagogical knowledge for active-learning instruction in large undergraduate STEM courses. This framework displays the collective ideas of participants as they analyzed the effectiveness of active-learning lessons in large undergraduate biology courses. There are seven components of pedagogical knowledge, some of which have subcomponents. Boxes with a dashed outline indicate video-specificity. Arrows indicate relationships among components that participants described

**Fig. 3:**
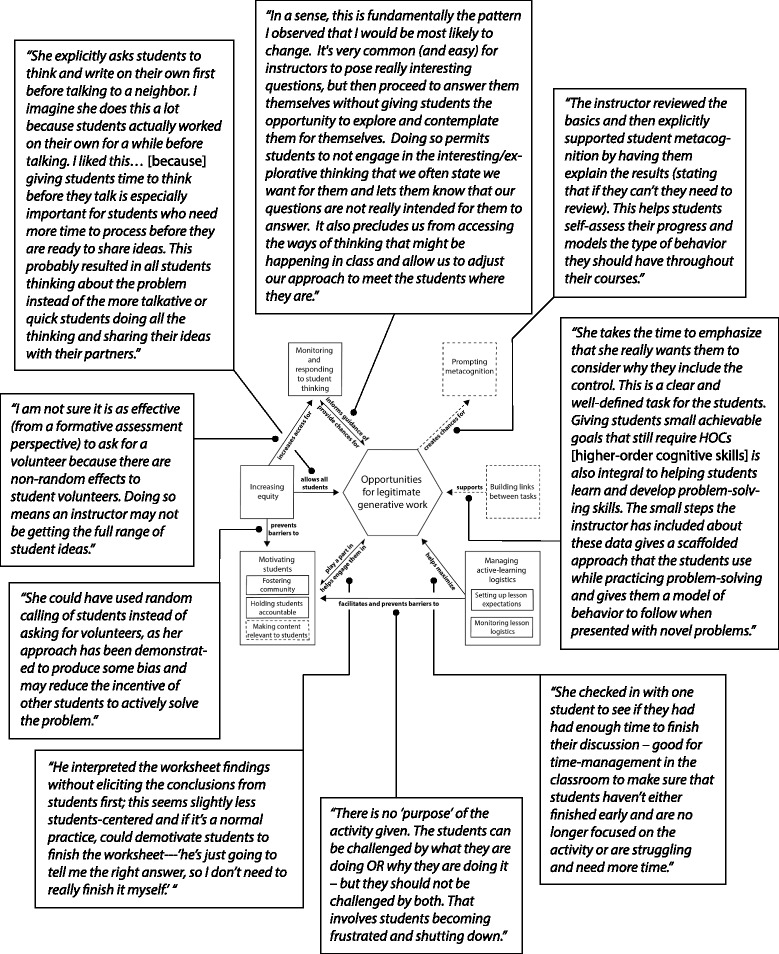
Quotes illustrating the connections between components of the framework of pedagogical knowledge for active-learning instruction. Each connection (arrows within the framework at the center of the figure) is linked to an illustrative quote that provides insight into participants’ thinking about how the components are related

The framework is structured as a box-and-arrow diagram with the components organized around a core component, which is depicted as a hexagon to further distinguish its centrality to pedagogical knowledge (Fig. 2). Throughout the paper, we begin by describing the core of the framework and then move around the core in a clockwise fashion, starting from the pedagogical component depicted on the bottom left. Subcomponents, which are closely related parts of a component, are represented as smaller boxes within boxes. Two components and one subcomponent were video-specific, meaning participants primarily discussed them in relation to a single video. We differentiate these with dashed boxes instead of solid boxes to indicate that these components are more tentative. The arrows between the pedagogical components communicate directionality of relationships among pedagogical components and have brief labels describing the relationships. These relationships are illustrated with quotes from participants in Fig. 3. Two arrows are double-headed, which indicate a close, reciprocal relationship between two components. We opted for one double-headed arrow rather than two separate arrows to emphasize that it was hard to distinguish directionality in participants’ thinking about these two components. Rather than one component clearly impacting another, some participants seem to see the two components as interacting in a positive feedback loop, with each component facilitating the other. Single-headed arrows indicate that participants primarily discussed a unidirectional relationship among the two components.

*Opportunities for legitimate generative work* form the core of the framework because it was our guiding definition of active-learning instruction and was woven throughout the thinking of our participants. Participants discussed this more than any other component and linked it directly to every other component. Participants discussed six additional pedagogical components that each relate to the core of the framework (Fig. 2). *Motivating students* includes fostering the course community, student accountability, and the relevance of the content to students’ lives. These impact whether students choose to do legitimate generative work during class time and are positively impacted by opportunities for generative work. *Increasing equity* in the classroom can help avoid barriers to student motivation to work and also allows all students the same opportunities to engage in legitimate generative work. Additionally, equal opportunities for students to participate give the instructor more representative information about student thinking. *Monitoring and responding to student thinking* during class time allow the instructor to make decisions based on where student thinking is at the moment. Opportunities for legitimate generative work create access to student thinking, and insights gained from this access allow instructors to modify instruction to improve student learning opportunities. Engaging students in generative work creates opportunities for *prompting metacognition*, which involves instructors explicitly guiding students to reflect on their own thinking and learning. *Building links between tasks* facilitate legitimate generative work by building challenging tasks upon what students learned previously and by making connections among tasks explicit. *Managing active-learning logistics* involves setting up a lesson so that students know what is expected of them and have enough time to meet expectations and then monitoring lesson logistics and responding appropriately. Managing the logistics of an active-learning lesson helps students engage more effectively and efficiently during class time and facilitates and prevents barriers to student motivation to work during class time.

### Descriptions of framework components

We provide an overview of participants’ thinking about each component, as well as assumptions that may underlie this thinking. These assumptions are tacit, meaning that an individual may not realize they possess the idea. We describe specific instructional practices that participants saw as contributing to each pedagogical component. We also summarize the connection of each component to other components both in the text and in Fig. 3. We draw extensively on the writing of our participants to describe each component, using quotes to reveal how instructors think about active learning in large courses. All texts in quotations were written by a participant unless it is described as being said by an instructor in a video clip used in the lesson-analysis survey. We edited for grammar only and occasionally included our own words in brackets within a quotation to add clarity. Quoted statements within a single sentence are not always from the same participant because we aimed to capture the words of many different participants. However, we always endeavored to retain the full meaning of participants’ sentences. Importantly, some participants’ ideas align with education research, and others lack the same evidentiary basis. Thus, we caution readers against using the words of these participants as advice for teaching without consulting education research and theory. The discussion addresses key knowledge used by our participants in light of existing literature.

#### Opportunities for legitimate generative work

Participants noticed and valued opportunities students had for generative cognitive engagement and engagement in legitimate scientific practices. They explained that students learn more when they are responsible for cognitively engaging in challenging work during class time. As they responded to the video clips of active-learning lessons, participants considered the level of cognitive engagement required by the tasks students completed and how the instructor facilitated student work during class time so that students had the chance to “construct their own knowledge about a topic.”

Participants evaluated the type of problems or tasks instructors asked students to complete. They explained that problems that ask students “to use their own logic” and that emphasize “providing reasoning rather than coming up with the correct answer” will “result in more sophisticated answers or arguments from students.” They praised tasks in which students had to “reach or extend their understanding a little beyond what was explicitly taught,” “defend their thinking,” “support their argument,” and take responsibility for “weighing the evidence.” Some participants also emphasized the value of collaborating to complete a task. They saw working in small groups as providing the chance for students to practice their “ability to communicate their understanding,” “learn something from their peers,” and “vet their thinking.” Some participants thought carefully about whether the cognitive engagement in small group work was interactive. For example,

I would give a little more time for discussion. My experience is that in one minute, both partners do not have enough time to share, or for there to be any challenging of ideas. I usually hear one student explain, the other one nod, but no real discussion.

One type of generative work participants noticed was opportunities students had to “engage in the practice of science.” Participants complimented tasks that asked students to “write down an explanation for experimental results” and “analyze new data and interpret it,” especially when “students were given real experimental data to analyze.” They valued students “learning about appropriate experimental design,” including struggling to think about “what controls were used and why there were important.” Participants explained that practicing these skills was important for “understanding the nature of science” and because it “trains students to think like a scientist.”

Participants also noticed the decisions instructors made as they facilitated class time and whether these decisions held students responsible for doing generative work throughout class time. Instructors must make choices about how to respond to students’ questions. Participants appreciated instances in which an instructor “resists giving the answer” and going into “explainer mode” and instead “keeps the onus of learning focused on the student.” Participants suggested that “probing the students with additional questions” can “help students arrive at the answer rather than telling them the answer.” The benefits of asking follow-up questions go beyond facilitating student learning. They also help “clarify a student’s thoughts for both the instructor and the students” and help the instructor “get a feel for common questions/misconceptions to help guide the class discussion later.”

Another way to respond to a student’s question is to direct “students to engage with each other to talk through the questions.” Prompting “confused students to seek guidance from peers” allows students to hear other perspectives and practice explaining their own thinking and also provides a chance for a “neighboring student to check their understanding.” This approach encourages students to continue to do generative work, rather than relying on the instructor’s thinking, as explained by this participant,

If an answer comes out of an instructor’s mouth, the student assumes it’s correct and just write it down. They’ve learned very little. If a students if forced to listen to another student’s explanation and decide if it is reasonable or not, then the student is using much higher-order cognitive skills in the exercise.

In addition to fostering “deeper learning,” opportunities for legitimate generative work can foster community among students. Some participants reasoned that a “peer explaining the concept to the student is less threatening to the questioning student than if the professor answered the question.” Giving students the chance to answer each other’s questions may also help students “become more comfortable with their classmates” which “increases participation and engagement” over time. One participant explained that “referring one student’s questions and observations to another student’s” can help students “start to see themselves and other students as colleagues with expertise in the science of cell biology.”

Participants also noticed how instructors facilitated the wrap-up of an active-learning task. One way to keep the responsibility for learning on the students is to “ask the students to share what they thought could be concluded from this activity.” Discussion following a task can address misconceptions that arose as students worked. Allowing multiple students to contribute to this wrap-up draws on “expertise among students about why these are wrong or at least why they think they could be right.” This provides additional opportunities for students to practice explaining their reasoning and sensemaking, which could be “a more powerful way to increase student learning.” None of the videos showed this, so these comments discussed a missed opportunity. One participant said,

In explaining the rationale, the instructor again took over on the explanation instead of relying on students to fill in the gaps. Several students could have constructed a complete response for the class instead.

#### Motivating students

Placing more responsibility on students to engage cognitively during class time increases the effort they have to expend during a class period and therefore increases the motivation students need to fully participate (Sinatra et al. 2003; Leonard et al. 2014). Therefore, achieving learning objectives in an active-learning course depends on student motivation to do the work necessary to learn. Participants paid attention to three ways of motivating students: fostering community, holding students accountable, and making content relevant to students.

##### Fostering community

Participants evaluated whether students in the video clips felt comfortable and like they belonged in the classroom. Underlying these comments was the assumption that instructor behaviors during an active-learning lesson influence students’ feelings and that these feelings affect students’ willingness to work during class time. Participants revealed different student feelings they consider in active-learning lessons. Some participants expressed a desire for students to “feel like they are important parts of classroom community” and to feel a “sense of belonging in the classroom.” Others focused on instructor behaviors that would communicate to students that “they are known” and “valued” by the instructor and that the instructor “cared about their learning process” and is “on the students’ side and there to help them.” Participants also observed whether a course seemed to be “safe” and “welcoming,” thus making “students feel comfortable” contributing their ideas and interacting with the instructor.

Participants described several instructor behaviors they saw as important for fostering community. First, they praised instances when an instructor used a student’s name. Knowing students’ names invites students “to participate because they feel like they are known and they belong.” It also serves as “real data showing they are valued” and can communicate “that we know them and care about them” and their engagement in the learning process. A second instructor behavior that participants saw as promoting a sense of community occurred during a whole-class discussion when an instructor explicitly asked for a volunteer who had not yet spoken that day to share their thinking. Participants reasoned that this can make the students feel like “they are important parts of the classroom community” and “that [the instructor] cares about everyone’s learning and not just the top students.” A third instructor behavior that can “engender a sense of community” is walking “away from the front of the room,” “circulating the room,” and “weaving through the aisles.” Walking among students allows for “interacting one-on-one with students in different parts of the classroom” and can communicate “concern for student learning.”

Participants praised opportunities students had to work alone and then discuss their thinking with their peers because they expected it to increase students’ willingness to do generative work during class time. Participants explained that the chance “to think about their answer” alone helps “those students who need a little more time” to have “more opportunities to become part of the discussion.” The chance to “formulate that answer verbally” and “bounce ideas off each other” in small-group discussions creates a “safe” and “non-threatening” setting before “reporting to the entire class in a more ‘high-stakes’ way.” This approach provides students with the “courage to speak out” during a whole-class discussion. This participant explained their thinking about the relationships between opportunities for interactive cognitive engagement, student comfort, and student learning,

She also allowed students to share their thinking with peers. This affords them the opportunity to vet their thinking (practice their ability to communicate their understanding) in a ‘safe’ setting. We know that talking through an ill-formed or complex concept can promote cognition. As we’re speaking, we are also linking new ideas to existing ones and refining our understanding. Doing so with peers in a ‘safe’ context enables a more authentic version of the process compared to being called on directly by an instructor without time to think/process/practice.

##### Holding students accountable

Participants reflected on how to hold students accountable for engaging in legitimate generative work during class time. Two underlying assumptions appeared in participants’ thoughts about this. First, some participants assumed that students would be more likely to work if they earned course credit (i.e., “points”) for doing so. Second, some participants assumed that students would want to avoid being perceived by the instructor and/or their peers as unprepared or unengaged. Notably, many of the instructional practices participants described as holding students accountable are also described as fostering community. Most participants who discussed these practices did not provide both rationales, indicating that active-learning instructors may value the same instructional practices for different reasons.

Participants considered how to hold all students accountable for “actually thinking about the question and providing an answer.” “Calling randomly” on students is one approach to help “ensure that students know that everyone is expected to speak and contribute.” If the instructor tells the students that only volunteers will be asked to share, “many students will feel that it is safe to check-out because they know that they will never volunteer.” Other participants did not advocate for randomly calling on students. One participant said they would “put students into groups (even in a lecture hall) so I can call on them.” Another proposed that students need time to talk to their peers prior to the random call.

Circulating the classroom and interacting with students engender a sense of accountability in multiple ways. Students may want to prevent the instructor from thinking they were not working and “it will be obvious if they are not working while the instructor is circulating.” Circulating is also an opportunity to “ask a random student what they think the answer is” because students “will want to be ready with an answer” if they think the instructor might ask their opinion. Using a student’s name when the instructor stops to talk to them may make them feel like the instructor is paying attention to “whether or not they are engaged.”

An instructor can also hold students accountable by requiring each student to turn in their own work. Students can turn in a written answer or submit their answer using a classroom response system. These strategies can prevent students from relying on peers or the instructor to do the cognitive work. In addition to being motivated to earn course points, students may be motivated in these situations because they “know that the instructor might read how they answered the question.”

##### Making content relevant to students

Participants also discussed the role of relevant and interesting content in motivating students. Participants explained that students would be more likely to pay attention, stay engaged, and work “if they see the relevance or importance” of a topic to their own lives. Instructors can accomplish this in multiple ways. Using examples that students can “identify with” and “make sense of from their own experiences” can be “more powerful” than “relying on the instructor’s interpretations or examples.” In addition, making the material interesting to students “acts as a hook that pulls people in” and can help hold students’ attention. One participant explained the relationships they saw between making content relevant to students and student learning this way,

The instructor used interesting/provocative questions linked to common misunderstandings to frame the instruction. I assume this is a lesson that is intended to show how phylogenies can be used as evidence for or against existing hypotheses. The question that sets the context is ‘did humans evolve from chimps?’ This is a common misunderstanding that gets repeated in media and lay contexts, so it is likely to be familiar to students and is a great way to engage students for the purposes of instruction about how to use phylogenies. The ‘effective’ part being, capturing student attention and interest with something that may be familiar in order to expand upon their knowledge by introducing something unfamiliar (how to read a phylogeny).

#### Increasing equity

Active-learning instruction in large college courses raises concerns about equity. These classrooms afford a variety of opportunities for student participation, including individual thinking and writing, small-group collaborations, and whole-class discussions. A student’s opportunity to fully engage in these opportunities can depend on the behaviors of peers and the instructor. Participants discussed how differences between students have the potential to create inequitable learning opportunities. While some participants discussed equity in relation to learning, others seemed to see equity as inherently valuable. Our participants focused primarily on equity in whole-class discussions, so we will discuss this first, followed by equity in small-group and individual work.

Participants thought about equity when they considered how instructors asked students to share their thinking during whole-class discussions. The choices an instructor make when choosing representatives to share their ideas has the possibility to produce bias in who responds to instructor questions, as well as who is heard. Two of the lessons included an instructor asking for student volunteers to share their thinking with the instructor and other students. The instructors in these lessons asked for volunteers in different ways. Participants felt one tactic hindered student inclusion, while the other encouraged inclusion. In one video clip lesson, students had worked in small groups, and then the instructor told the students, “I would love to hear from someone who hasn’t had a chance today. Who’s been thinking about the first question and would be willing to read or share what they have for this first part?” Participants praised this instructor for aiming to elicit a response from a new student, stating this would include students who were “less vocal” and “not just the ones who are most inclined to speak up in class.” They saw value in “adding more voices to the classroom” and encouraging “diverse participation.” Some also reasoned that equity is important because it influences student motivation. One participant described these longer-term effects on student participation that could result from this approach,

If you begin to let the same students always answer questions, the students will pick up on this and they will not even try to offer answers because they know that students X and Y always answer the questions.

In another video clip, the instructor prefaced an activity by informing students she would ask for a volunteer, “So talk to each other, and then I want someone to volunteer why it is that the answer to that is no.” Participants thought this approach could hinder equity and suggested using “randomized call” instead of asking for volunteers. They expected that volunteered answers would be “disproportionately from men” and would lead to the “same small group of students dominating whole-class discussions.” Participants expected that randomly selecting students to share their thinking would result in “a greater diversity of students reporting answers in class” and would support “a whole community of students, rather than just the one loud student up front.” Hearing from a small number of students may limit an instructor’s ability to effectively monitor and respond to student thinking in real time.

Students may also experience inequitable opportunities in small-group discussions. Interacting with peers during small-group work may be more challenging for some students than others due to social dynamics. Our participants commonly discussed how students with different personality characteristics might experience group work differently. Most commonly, participants focused on the inclusion of “shy” or “quiet” compared to students who are “most inclined to speak up in class” and how that was influenced by the instructor. Participants valued think-pair-share activities implemented with time for individual thinking, talking in a small group, and sharing among all students. This approach provides “introverts” or “individual thinkers” with “quiet time to sort through their own understanding of the exercise/question or find the place at which they get stuck before the room gets noisy.” Allowing time for students to think before they talk to each other allows all students the opportunity for generative cognitive engagement “before being bombarded with the reasoning of others.”

Our participants rarely (or never) considered other differences between students that might influence social dynamics and make group work more fraught, including students who identify as LGBTQIA, have a learning disability, have an autism spectrum disorder (ASD), are first-generation students, are international students, or are non-native English speakers.

#### Monitoring and responding to student thinking

Participants viewed student thinking as central to an active-learning lesson. They assumed that knowledge is constructed by the learner and that the goal of instruction is to help students recognize and modify their own thinking. Providing students with opportunities for generative work in class time allows the instructor a way to access student thinking. Instructors cannot necessarily predict which ideas will arise during a task and how student thinking will change as they engage in generative work. Therefore, facilitating opportunities for generative work often requires responding in real time to student thinking. Doing so requires carefully monitoring student thinking.

When students do legitimate generative work during class time, it allows the instructor to “gather intel about student thinking.” This access to student thinking helps instructors “see what [students’] misconceptions are at the point” and respond accordingly. Participants noted that eliciting student thinking was necessary to determine a starting point for instruction, to gauge the effectiveness of a task, and to respond in real time to student confusions or difficulties. They discussed ways to gather information from all students, including polling via clickers or raised hands, circulating while students work to eavesdrop and invite questions, and collecting the work students produce. Participants saw value in starting a discussion “by posing a question” and “polling students” in order to establish “what they understood already.” Polling can also provide opportunities to determine who agrees with a line of “student reasoning” and to see “how many minds changed.” It is important for the instructors to “hear a range of responses” so an instructor can “better calibrate” where students are in their understanding.

Participants discussed how feedback on student thinking “gives the instructor real-time insight into how to proceed” with their teaching. Instructors can elicit student thinking to “tailor the teaching to review the topic if necessary.” “Asking a student to share their reasoning with the entire class” allows the instructor to “modify or refine” student thinking in their own explanation to the students to ensure that “all students are exposed to a good example of reasoning.” This participant described how they would gather and use feedback from students,

I would have walked up the sides of the room at a minimum and listened to a few groups. Often when students see an instructor coming they will ask a question. This gives me a bit of insight about where they students are, and may help to guide the report-out once the groups have come up with support for their answer.

#### Prompting metacognition

Metacognition, which is defined as awareness and control of thinking for the purposes of learning, can greatly improve student learning. Most students need an external indicator, such as feedback, in order to evaluate their own knowledge and make necessary changes to their approaches to learning (Dye and Stanton 2017). Active-learning classrooms create new opportunities for students to get “real-time feedback on their in-progress learning,” which can help students “recognize if they do or do not understand a topic.” Participants praised instruction that “helps students to think objectively about their own learning.”

Participants explained that a variety of generative tasks can prompt metacognition. First, “by asking students to write on their own, the instructor is making the students recognize what they actually know and do not know.” Second, asking students “to explain their answers to their group” provides “additional opportunities for students to check their understanding.” Third, after hearing a student explain his or her thinking, an instructor can provide feedback to all students so that students can “reflect on how they were approaching the question posed” and “gain insights about their own knowledge.”

Students may not possess skills that allow them to be independently metacognitive and therefore may require explicit instruction to promote metacognitive reflection. In one lesson analyzed by participants, the instructor asks students to write out their understanding of a concept covered previously. The instructor in the video said,

What I want you to do is explain the data in lane 5, which you’ve looked at before, so this is a great opportunity to monitor your understanding. If you’re like, ‘Yeah, I got it in breakout’ and then you can write it here, you are golden. If you’re like, ‘I can’t actually write it,’ then you know you need to go back to it.

Participants thought this type of guidance “encourages students to reflect on what they know” and ultimately enables them to concentrate “studying and review efforts on material they struggle to explain.” It was important for the instructor to tell the students “how to interpret their performance on an exercise” to help them “modify their studying accordingly.”

Participants explained that this instructor’s explanation to students trains them “to think this way on their own.” One participant explained how she talks to her own students about the study approaches that will help them develop conceptual understanding. She aims to model a way to think about their own learning that they might not have considered previously,

At the beginning of the video the instructor mentions that “even though we have talked about the results in Lane 5, see if you can explain these results in writing.” I talk with my students about this all of the time. I tell them that your brain is really good at “tricking you” into thinking that you understand something, when really all you have achieved is fluency with the words in the questions. For example, a common complaint amongst students is that “they felt like they understood everything in class or when they were reading it, but when they got to the test they couldn’t answer any of the questions.” We talk about how what causes this problem is that students are becoming more fluent with the language of biology and their brains are confusing this with understanding. So, to determine if they are fluent or if they actually understand a concept, they must attempt to answer a question in WRITING or explain it orally to a friend. Only after doing this will they really be able to assess whether they understand the topic.

#### Building links between tasks

Participants valued instances when tasks and problems clearly built upon each other. They saw this structure as supportive of generative cognitive engagement and learning more broadly. Reminding students “they have seen this before” helps “activate prior knowledge.” Tapping into “students’ prior knowledge” helps students “organize what they know and get ready for new material.” Building on what students learned previously provides “space for students to process and extend that thinking beyond what was explicitly taught” and “allows students to use higher-order thinking—analyze and evaluate—to broaden their understanding.”

Participants praised an instructor who explicitly pointed out links between prior and current tasks because the students “make connections to their previous learning and add to an existing conceptual framework.” Making links explicit may also help students avoid frustration and build confidence. “A chance to review something they have done before” can help remind students “that they should have a grasp on that particular material.” This, in turn, can help students recognize what they have already accomplished, “increase confidence” for working on challenging new problems, and prevent students from “being overwhelmed.”

#### Managing active-learning logistics

Asking students to engage in generative work in a large course raises different logistical challenges than lecturing. Instructors cannot perfectly predict how students will interact with a task, including whether they will understand what they are being asked to do and how much time they will need to do it. The participants noticed what the instructors did to set up student work, including making instructions and expectations clear to students and giving them enough time to meet those expectations. They also noticed how the instructors monitored lessons and made adjustments to make sure that students had adequate direction and time.

##### Setting up lesson logistics

Participants noted that instructors help students work effectively and efficiently and avoid frustration when they provide clear instructions for how to complete a task and make expectations for what happens during class time clear. When an instructor provides “clear instructions and an itinerary of what will happen next,” students know what is expected of them. This “helps students structure their time throughout the activity” and may make a lesson more efficient by helping students get to work “as soon as they [are] asked to.”

Instructors can set norms for what is expected of students early in the semester. Participants concluded that “students clearly know the drill” and feel comfortable with group work when they “immediately started to discuss the topic.” A few participants noted that these behavioral expectations are often set early in the semester because “in-class activities work best if their instructor begins to use them during the very first class period and then consistently uses them throughout the semester.”

Clear instructions and expectations allow students to have more “meaningful time on task” instead of getting “bogged down in wondering what the instructor actually wants them to do.” Energy and time that students spend figuring out the logistics of a task is energy and time they are unable to spend working to understand concepts and develop skills, and therefore negatively impacts learning. For example, this participant explained,

[The instructor] also appears to be highly organized with a well thought-out plan for the activity - this contributes to efficacy by making the process orderly and not adding procedural confusion that can compete with the learning process - i.e., we only have so much bandwidth at a time; if we have to think hard about ‘how/what’ to do procedurally, we probably aren’t thinking hard about the concept underlying the activity.

Participants thought that explaining the reasoning behind a problem or task to students would keep them motivated to work. Explaining to students “why they were doing the exercise” helps students “have a clear goal in mind” for what they should accomplish. Instructions about “what they were expected to get out of the activity” helps keep “students focused on the task at hand and accountable for what they are supposed to be learning.” Explaining why they are engaging in a task may also drive “more students to take the activity seriously.”

Setting expectations and giving clear instructions for a task set the stage, but students also need time to work to meet those expectations. Participants commonly paid attention to whether students had enough time to complete a task. Giving too much time runs the risk that “students have moved on to non-class topics,” but “cutting off the activity while students are actively working on it may make the activity less effective.” Participants explained that students need “a lot of time to do something they are not used to doing,” that ample time to work “promotes deep thinking,” and that “the more students talk, the more they have a chance to learn.” Determining how much time students need and whether instructions are clear requires real-time monitoring.

##### Monitoring lesson logistics

Participants noticed how instructors circulated the classroom to monitor the logistics of a lesson, including how they determined the timing and responded to student confusions about what they were supposed to be doing. These logistics can indirectly influence student learning by affecting how students spend their time. Participants explained that active-learning lessons may require instructors to “adapt quickly to questions and uncertainty in the activity.” Moving throughout the classroom to “check in with students about how the activity is going” allows instructors to be responsive. Checking in with students can help an instructor determine “whether or not he had given sufficient instruction” and whether “pacing was fitting the students’ needs.” When an instructor recognizes that students are confused about how to begin a task, he can “answer these questions for the whole class” in order to “allay confusion for everyone, not just the student who asked the question.” Responding promptly “helps students move through the procedures more quickly and focus more on the main biological concepts.” Closely monitoring student progress on a task to determine how much more time they need prevents “students from rushing or waiting too long.” This participant explained how instructors can employ more sophisticated approaches to monitoring timing as they get to know their students better,

At the end, she checks in with a group of students to see if they had enough time to discuss their ideas. If you know the relative pace of different groups and you strategically choose which group you ask, then this can give you reliable information about when to cut off discussion.

### Variation across participants

**Fig. 4:**
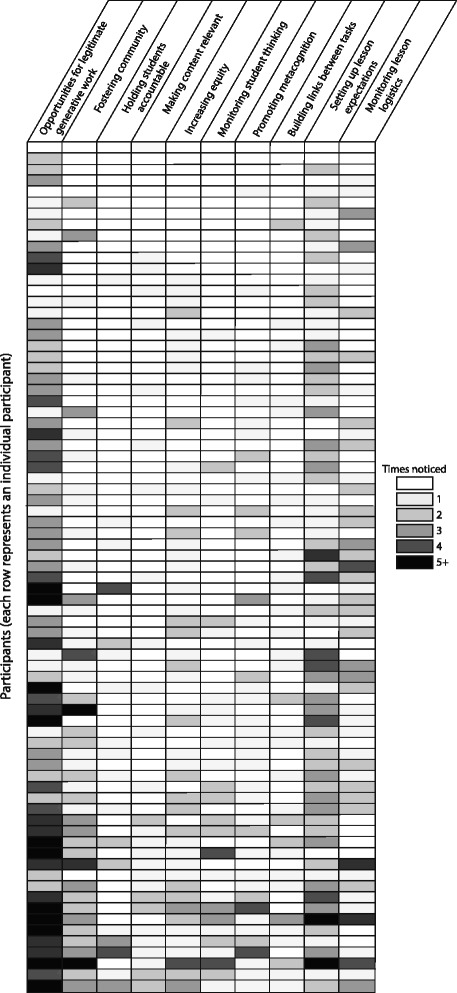
Heat map of the number of times each participant noticed each component and subcomponent in the framework. Each row of the heat map represents an individual participant. Participants are ordered from the lowest to the highest number of components plus subcomponents noticed. Each box within a row is shaded to indicate the frequency with which that participant noticed that component or subcomponent. Darker shades correspond to higher frequencies. Each column represents a component or subcomponent, and the columns are ordered as they are described in the results. Indented column headers indicate subcomponents

Most participants did not discuss every component in the framework (Fig. 4). In fact, only two participants discussed every component and subcomponent, and only seven discussed every component. On average, participants discussed 5.4 (SD = 2.0) parts of the framework out of 10 total components and subcomponents. Almost everyone paid attention to opportunities for legitimate generative work and to setting up lesson logistics, and participants commonly addressed these more than once across their analyses of three lessons (Fig. 4). The components that participants most commonly omitted were holding students accountable, making content relevant to students, monitoring and responding to student thinking, and building links between tasks. These data highlight the fact that the framework of pedagogical knowledge is built on the collective thinking of our participants, rather than any one individual’s thinking.

## Discussion

This in-depth look at the thinking of 77 participants generated a framework of pedagogical knowledge for active-learning instruction in large biology classes. Pedagogical knowledge has been underemphasized in teacher knowledge research in recent decades. The diversity of pedagogical knowledge employed by our participants suggests this teacher knowledge base deserves renewed attention, especially as it relates to active-learning instruction in large undergraduate classes. The comprehensive framework we present is firmly grounded in the thinking of life sciences instructors and should be treated as a hypothesis that needs additional testing and refinement. It lays a groundwork for determining what knowledge is most important to effective active-learning instruction and in what instructional contexts. We discuss implications of this framework for preparing and supporting active-learning instructors in the undergraduate STEM and for future research on teacher knowledge for undergraduate instruction. We also highlight limitations.

Active-learning instructors in this study displayed knowledge of principles of how people learn that are grounded in educational theory. Principles of how people learn are overarching ideas about the nature of knowledge and learning. Our data do not reveal the degree to which instructors were familiar with education literature or specific learning theories. However, participants considered students’ cognitive, affective, and metacognitive thinking, and each of these is grounded in theories of how people learn (Vermunt 1996). Cognitive thinking deals with processing content to learn, affective thinking involves coping with the feelings that arise during learning, and metacognitive thinking includes regulating cognitive and affective thinking (Vermunt 1996). Therefore, we hypothesize that teaching professional development for active-learning instruction that does not help instructors plan for the cognitive, affective, and metacognitive dimensions of active learning will fall short of promoting effective instruction.

In discussing the cognitive dimension of active-learning instruction, participants embraced constructivism as a principle of how people learn. More specifically, the ICAP framework was useful for characterizing participants’ thinking. Participants widely considered whether students were engaged in constructive work, and some discussed the opportunity for interactive work, noting that students can learn from explaining their thinking to peers and building on the thinking of their peers. We propose that the ICAP framework could be a useful tool for helping instructors learn to distinguish generative work from active or passive work, and therefore fostering a constructivist rather than a transmissionist view of learning (Chi and Wylie 2014). The prevalence and dominance of constructivist views in our sample suggest that preparing and supporting active-learning instructors require creating opportunities for them to reflect on and reject the idea that students can develop deep understanding by “receiving” content as they listen to lectures.

Participants also considered whether the work asked of students gave them opportunities to engage in the practices of science. This suggests that some important aspects of a sociocultural perspective of learning have infiltrated the thinking of these instructors. Other important views associated with this perspective of learning, such as learning as enculturation and knowledge as participation in a community of practice, were less obvious in the thinking of our participants. It is possible that adopting constructivism as a principle of how people learn comes more easily to college biology instructors than adopting a sociocultural perspective. Yet, a sociocultural perspective that views learning as something more than knowledge acquisition may be better aligned with the true benefits of active-learning instruction for undergraduates in STEM. Future work should continue to focus on the learning theory that instructors need to embrace to use active-learning instruction effectively.

In addition to thinking about students’ cognitive work in class, participants showed knowledge of the role of affective learning as they evaluated active-learning lessons. About 74% of participants addressed student motivation at least once, and 88% discussed motivation or equity. Participants paid particular attention to instructor behaviors they expected to increase or hinder students’ sense of belonging to a community (i.e., *fostering community*). There is compelling evidence that attending to students’ feeling in the classroom is important to effective active-learning instruction. A literature review revealed that about half of the instructional practices demonstrated to improve student achievement in active-learning instruction in large undergraduate STEM courses focused on student motivation (Eddy et al. 2015). Furthermore, a sense of belonging contributes to persistence in STEM for students from underrepresented groups and can positively influence the academic motivation, academic achievement, and well-being of all students (Trujillo and Tanner 2014). An in-depth study of instructor talk, which is any language used by an instructor that focuses on creating the learning environment, found that about 70% of the instructor talk used by two co-teachers was related to the instructor-student relationship or establishing classroom culture (Seidel et al. 2015). This instructor talk can increase student’s perception of the closeness of the instructor-student relationship and contribute positively to learning and persistence (e.g., Witt et al. 2004; Seidel et al. 2015). Yet, as Seidel et al. (2015) point out, teaching professional development has not often emphasized the importance of attending to students’ affective thinking to improve learning. We hypothesize that preparing college STEM instructors to seriously consider student affect is as important to active-learning instruction as considering the cognitive work asked of students.

In order to best support active-learning instructors in considering student affect in active-learning courses, we urgently need more investigation of the experiences of diverse students. An exploratory study of the experiences of LGBTQIA students in a large active-learning course found that these identities were more salient in an active-learning course because of increased interactions with other students during group work (Cooper and Brownell 2016). This study also discovered that some LGBTQIA students felt uncomfortable working in groups, and feeling uncomfortable may negatively influence students’ learning. For example, students who felt uncomfortable in their group achieved worse content mastery than students who were comfortable in their group in an active-learning class (Theobald et al. 2017). On the other hand, an active-learning course may provide better opportunities for LGBTQIA students to come out and find similar others than traditional lecture courses, which also has important benefits (Cooper and Brownell 2016). Systematic investigations of the experiences of different groups of students in active-learning courses are relatively sparse but will be important to enriching our understanding of how to maximize the experiences and learning of all students. Much like the research community, our participants did not seem to think much about the experiences of students with different racial, ethnic, socioeconomic, and LGBTQIA identities. Thus, increasing our sociocultural awareness as researchers may facilitate sociocultural awareness among instructors, ultimately contributing to the diversity in STEM.

Participating instructors also demonstrated knowledge of the role of metacognitive thinking in learning. Metacognition involves recognizing what you do and do not know and taking control of your own thinking for the purposes of learning and is part of self-regulated learning theory (Dye and Stanton 2017). Metacognition is positively associated with outcomes (Wang et al. 1990), but undergraduates commonly lack the metacognitive knowledge and regulation skills they need to develop conceptual knowledge (e.g., Stanton et al. 2015). One video that participants analyzed featured an instructor prompting students to engage in metacognitive thinking, and this caught the attention of about half of our participants. The video specificity of this result makes it more tentative, but prior theoretical and empirical work suggests that metacognition is likely to be important when we ask students to engage in legitimate generative work. Therefore, we see value in future work to test the hypothesis that active-learning instructors are more effective at facilitating student learning when they possess and apply knowledge of how to explicitly facilitate students’ metacognitive regulation of their own learning.

Participant’s knowledge of principles of how people learn was augmented with practical knowledge of instructional approaches. Specifically, participants commonly discussed strategies for supporting positive student affect. Two instructor behaviors that participants considered important for supporting a sense of belonging and comfort in the classroom were using a student’s name and circulating the classroom. Learning the students’ names is suggested as a way to foster instructor-student relationships, but can seem like a daunting task for instructors of large classes. One recommended approach to aid in learning and using students’ names is name tents displayed by each student each day in class (e.g., Tanner 2013; Cooper et al. 2017). When asked why they thought it was important for an instructor to know their name, undergraduates in a large active-learning course echoed much of what our participants described as the benefit of knowing names; they felt more valued and cared about by the instructor, they felt more invested in the course, and they were more comfortable getting help from the instructor (Cooper et al. 2017). Additional research will need to investigate further the role of using students’ names in large active-learning courses and what other approaches can have similar positive effects on how students feel about the course. Learning names, even only half of the students’ names, may be untenable in classes with over 300 students, or for instructors teaching multiple large sections each semester.

Another instructional strategy commonly praised by participants should be possible in many active-learning courses—circulating accessible sections of the classroom as students engage in legitimate generative work. Participants saw this simple behavior as important for building relationships with students, monitoring student thinking, monitoring lesson logistics, holding students accountable for staying on task, and responding immediately when students need additional direction or clarification. This behavior, perhaps due to its simplicity, has not been the focus of the empirical investigation, yet it may be a hallmark of active-learning courses that the instructor leaves the front of the room as students work. We hypothesize that circulating the classroom is a behavior change that instructors can make easily and that this change might facilitate other instructional changes by providing better access to student thinking and by facilitating one-on-one relationships with students.

A third instructional approach that participants discussed was using a random call to ask students to share their ideas with the whole class. Participants expressed alternative perspectives, including the idea that random call helps foster equity by ensuring that everyone has an equal chance for their voice to be heard and the opposing idea that random call negatively influences the classroom community by making students anxious and uncomfortable. A common alternative to random call is to ask for students to volunteer to share their thinking. This can result in a gender bias in who participates because males are disproportionately likely to volunteer (Eddy et al. 2014). One potential impact of this gender bias is that students perceive that the most knowledgeable students in the class are male, even when this is not the case (Grunspan et al. 2016). This perception may negatively influence self-confidence among women and ultimately their persistence in STEM. Random call avoids gender bias in who is heard in the classroom and thus may indirectly influence persistence in STEM (Eddy et al. 2014). The random call may also increase the level of cognitive engagement of students. Using a random call to select groups to share the outcome of their small group discussions resulted in a higher proportion of discussions that contained exchanges of reasoning compared to asking groups to volunteer to share their ideas (Knight et al. 2016).

Though these studies indicate a positive impact of random call on student outcomes, they do not address participants’ concerns that students will feel uncomfortable and anxious. Classes in which students feel comfortable participating in class discussions have higher levels of participation in these conversations, so student comfort cannot be overlooked (Dallimore et al. 2010). A study of multiple instructors teaching the same course examined the effect of different levels of random call, which they referred to as cold-calling. The random call was significantly associated with students voluntarily answering questions. Additionally, students in classes using higher levels of random call reported a greater increase in their comfort level with participating in class discussions (Dallimore et al. 2013). The authors hypothesize that the chance to practice sharing ideas in class discussions, which occurs when a student is randomly selected to share, actually increases student comfort with sharing their ideas and thus makes them more likely to volunteer to answer questions in the future. These empirical findings run contrary to the sentiment that random call should be avoided due to student discomfort.

Beyond knowledge of principles of how people learn and practical knowledge of instructional strategies and behaviors, participants displayed knowledge about lesson management. The practical concerns of what it takes to plan and manage the logistics of an active-learning lesson in a large course have received little research attention, but we hypothesize that this knowledge is crucial to effectively execute a large active-learning course. The knowledge of how to provide clear instructions and sufficient time, and how to monitor to determine when students need more of either of these, is not particularly exciting, but a lack of this knowledge may undermine all other efforts to support students in engaging in legitimate generative work.

Notably, most instructors in our study did not reveal knowledge about every component of pedagogical knowledge, and there was a considerable variation in what knowledge instructors used (Fig. 4). This underlines the importance of addressing this question in future research—what knowledge do instructors need to effectively implement active-learning instruction in large undergraduate biology courses? One way to interpret our findings is that not all of these components of pedagogical knowledge are really necessary for effective active-learning instruction, and thus instructors did not draw on this knowledge in their lesson analyses. Another way to interpret our findings is that most active-learning instructors still have room to gain additional pedagogical knowledge that would positively contribute to their implementation of active learning. Additional research can help tease out which interpretation is more accurate. For now, though, we can conclude that the knowledge used by participants when they viewed the same example of instruction and responded to the same prompts varied considerably. It is therefore unreasonable to assume that all college biology faculty have the same pedagogical knowledge at their disposal. If pedagogical knowledge is important to the effectiveness of active-learning instruction, then teaching training and support for future and current college instructors will need to explicitly support the development of this knowledge base.

Pedagogical knowledge may also be important because it helps instructors develop other knowledge bases. Pedagogical knowledge facilitated the development of pedagogical content knowledge (PCK) among high school teachers who were teaching a topic for the first time (Chan and Yung 2015, 2017). These teachers relied on the idea that students have prior knowledge on which new learning builds, and knowledge of approaches to formative assessment to design questions to help them learn what students were thinking during class. The knowledge they used aligns well with the component of our framework called *monitoring and responding to student thinking*. What they learned by eliciting student thinking about the topic contributed to their PCK for teaching this new topic. In fact, an instructor who did not draw on pedagogical knowledge to the same degree did not develop as much PCK from his first experiences teaching this topic as an instructor who employed more pedagogical knowledge (Chan and Yung 2017). Thus, pedagogical knowledge was the key to developing PCK. We also wonder if the fostering community component of pedagogical knowledge in our framework could facilitate the development of knowledge of students by helping instructors get to know their students. Future research should investigate the interrelationships of these knowledge bases to better understand how they build upon each other.

### Limitations

There are several limitations readers should take into account when interpreting our results. First, the pedagogical knowledge revealed by instructors as they analyze lessons depends on the lessons themselves. We asked instructors to evaluate specific examples of instruction and used their evaluations to generate a framework of pedagogical knowledge that we hypothesize is generalizable beyond these lessons. The fact that our data include instructors’ analyses of three distinct lessons suggests that the components that were not video-specific are generalizable across active-learning instruction. Future work should test whether instructors rely on the same components of pedagogical knowledge and make the same connections among components when they analyze other examples of active-learning instruction in large classes, with special attention to the components that were video-specific in our study. For example, does making lesson expectations clear contribute to promoting metacognition? This connection between active-learning logistics and prompting metacognition was not apparent in our data, but maybe a connection that instructors make when different stimuli are used.

Second, this study was limited to college biology instructors. It is possible that different pedagogical knowledge is important in different STEM disciplines. We contend this is less true for pedagogical knowledge than for other teacher knowledge because pedagogical knowledge is content-independent. Additionally, the life sciences are very broad, and the topics in the videos analyzed by participants ranged from cellular biology to population biology, so we did not expect participants to be content experts for each of the three topics in the video lessons. Future work must test this framework of pedagogical knowledge across STEM disciplines, building on and refining this hypothetical framework.

Third, our research approach reveals knowledge that participants bring to bear when analyzing lessons taught by other instructors. The knowledge they use in their own teaching may differ in important ways from what was revealed by our work. Instructors’ contexts, beliefs, and short- and long-term goals may influence the knowledge they actually use to make instructional decisions in their own teaching (e.g., Schoenfeld 1998; Gess-Newsome 2015). Additionally, instructors likely integrate multiple knowledge bases, some of which are context- and topic-specific, to make instructional decisions, and our method of eliciting instructor knowledge is unlikely to have captured all of this knowledge. For example, instructors’ knowledge of their own student population may inform their specific pedagogical decisions, such as decisions related to student motivation. In the future, it will be critical to examine instructor knowledge used in planning and implementing active-learning instruction in their own courses. Such a research design would also lend itself to more directly examining the relationship between teacher knowledge and specific instructional practices, perhaps assessing instructional practices using published classroom observation protocols (e.g., Smith et al. 2013; Eddy et al. 2015). Our results lay important groundwork for such a study by identifying components of teacher knowledge that can the focus of investigations of instructional practices and specific knowledge teachers bring to bear in planning and using those practices. Video stimuli may continue to be important in these investigations but could be taken from an instructor’s own classroom (e.g., Alonzo and Kim 2016).

Fourth, the participants in this study are unlikely to be representative of typical college STEM instructors. Our sample also over-represents female instructors and under-represents racial and ethnic diversity, and these differences may impact the knowledge participants have constructed from their lived experiences. For example, it is possible that a more diverse sample of participants would have focused more extensively on the role of racial, ethnic, socioeconomic, and LGBTQIA identities in active-learning classrooms. Furthermore, all of our participants had tried active-learning instruction in a large course, and they had more experience with teaching professional development and discipline-based education research than we expect in typical instructors. It is possible that experience with education research and teaching professional development helps build knowledge that is important for effective active-learning instruction, such as knowledge of principles of how people learn. Future research will be crucial to understand how instructors develop knowledge important to effective active-learning instruction.

## Conclusions

Realizing widespread improvement in the preparation and diversity of STEM undergraduates requires reforming not just our instruction but also our preparation of undergraduate instructors. Active-learning instructors in this study drew on rich pedagogical knowledge. Instructors are not likely to gain such knowledge through training in their STEM discipline. Instead, college instructors likely need opportunities for legitimate generative work that helps them develop pedagogical knowledge for active-learning instruction. Future research is needed to better understand how to provide such learning opportunities for college STEM instructors. What is clear is that our current approach to preparing undergraduate instructors is unlikely to be successful in helping them effectively plan and implement active-learning instruction in large courses. This work contributes to the foundational knowledge we need to begin reimagining our philosophies and approaches to training and supporting undergraduate STEM instructors.

## Abbreviations

LGBTQIA: Lesbian, gay, bisexual, transexual, queer/questioning, intersex, asexual
NSF: National Science Foundation
PCK: Pedagogical content knowledge
PK: Pedagogical knowledge
STEM: Science, technology, engineering, and math
TPI: Teaching practices inventory

## Footnotes

1 A sociocultural perspective of learning is also referred to as a situative perspective. Alternatively, some refer to this as a participation perspective or metaphor (Sfard 1998; Mason 2007; Vosniadou 2007; Wegner and Nückles 2015).

2 Notably, the contrast between a cognitive and sociocultural perspective has been linked to the debate about whether PCK is a canonical and static body of knowledge (a cognitive view of knowledge) or emergent in the context of teaching (a sociocultural perspective) (Depaepe et al. 2013).
